# A Community Health Record: Improving Health Through Multisector Collaboration, Information Sharing, and Technology

**DOI:** 10.5888/pcd13.160101

**Published:** 2016-09-08

**Authors:** Raymond J. King, Nedra Garrett, Jeffrey Kriseman, Melvin Crum, Edward M. Rafalski, David Sweat, Renee Frazier, Sue Schearer, Teresa Cutts

**Affiliations:** Author Affiliations: Nedra Garrett, Center for Surveillance, Epidemiology and Laboratory Services, Centers for Disease Control and Prevention, Atlanta, Georgia; Jeffrey Kriseman, State of Tennessee, Nashville, Tennessee; Melvin Crum, National Center for Injury Prevention and Control, Centers for Disease Control and Prevention, Atlanta, Georgia; Edward M. Rafalski, BayCare Health System, Clearwater, Florida; David Sweat, Shelby County Health Department, Memphis, Tennessee; Renee Frazier, Common Table Health Alliance, Memphis, Tennessee; Sue Schearer, Methodist Le Bonheur Healthcare, Memphis, Tennessee; Teresa Cutts, Wake Forest School of Medicine, Winston-Salem, North Carolina. At the time this study was initiated Dr King was a Public Health Informatics Fellow at the Centers for Disease Control and Prevention, Atlanta, Georgia, and Dr Cutts and Dr Rafalski were at Methodist Le Bonheur Healthcare, Memphis, Tennessee.

## Abstract

We present a framework for developing a community health record to bring stakeholders, information, and technology together to collectively improve the health of a community. It is both social and technical in nature and presents an iterative and participatory process for achieving multisector collaboration and information sharing. It proposes a methodology and infrastructure for bringing multisector stakeholders and their information together to inform, target, monitor, and evaluate community health initiatives. The community health record is defined as both the proposed framework and a tool or system for integrating and transforming multisector data into actionable information. It is informed by the electronic health record, personal health record, and County Health Ranking systems but differs in its social complexity, communal ownership, and provision of information to multisector partners at scales ranging from address to zip code.

## Introduction

Health begins in the community; it is a product of where we “live, learn, work, and play” (1,2). Chronic diseases are responsible for most morbidity and mortality in US communities (3). The chronic disease burden, disparities in distribution and determinants, and the information and interest of the sectors that can affect these factors converge at the community. Community refers here to the geographic community at the subcounty level. It is at this level that community health information can be exchanged to inform a portfolio of multisector interventions for preventing and controlling chronic diseases and their determinants (4,5). Despite this importance, access to community health information at the community level is limited (6,7).

Multiple sectors (community health stakeholders), including public health, health care, community-based organizations, government, health care payers, community members, housing, education, and business, are interested in reducing the burden of chronic diseases and improving community health. Such an effort requires multisector collaboration and information exchange to inform decision making and target action (6–12). Traditionally, these stakeholders have not consistently collaborated or exchanged information, resulting in an inefficient use of resources, duplicated efforts, and often limited impact (9,11).

Advances in the availability of information and technology may be leveraged to facilitate collaboration and information exchange. Each community health stakeholder has access to a subset of this information and technology and varying degrees of capacity to take advantage of either. To construct a picture of community health, these fragments of information and technology must be combined and the capacity to use them developed. Success requires that all stakeholders have relevant access to their communities’ information and the capacity to use it to aid decision making (6,10,12).

Events in the national health landscape, namely the Patient Protection and Affordable Care Act (ACA) and the Health Information Technology for Economic and Clinical Health Act (HITECH), are encouraging such an approach in an effort to improve the quality and reduce the cost of care and ultimately improve the health of populations in our communities (13,14). The problem, however, is that no framework or infrastructure exists for bringing multisector stakeholders and their health-related information together at the community level to improve community health. We outline a framework for developing a community health record to bring people, organizations, information, and technology together to document the health status of a community, identify needs and priorities, aid decision making, and evaluate population health interventions.

## Approach to Solving the Problem

Health-related information systems are used to aid decision making at individual, health system, county, and larger jurisdictional levels. These systems are vital to their end-users within the context and scale for which they were developed. Community health stakeholders also need a process and a system that build on these approaches and enable standardized exchange, integration, and transformation of data from these and other multisector systems into information to aid community health decision making.

At the individual level, clinical medicine increasingly uses electronic medical record (EMR) and electronic health record (EHR) systems to inform decision making (15–17). Patients can increasingly access their health care information using patient portals and personal health records, although, their use is limited (16,18,19). Relevant characteristics for each of these systems are presented in the Box.

**Box. Characteristics of Electronic Medical Records, Electronic Health Records, Patient Portals, and Personal Health Records**

**Electronic Medical Record (EMR) (** **15** **–** **17** **)**

Documents episodes of patient care in a single health care organization

Providers use to facilitate patient diagnosis and treatment, track patient data, identify patients needing preventive services, monitor a patient’s conditions, and improve care quality

Collects, manages and displays patient demographics, physician notes, laboratory and imaging test orders and results, and prescription orders and alerts

Information is owned by the health care provider or organization and shareable only within that organization

**Electronic Health Record (EHR) (** **15** **–** **18** **)**

Includes and expands on EMR functionality

Uses interoperability standards to securely exchange information within and between health care organizations

Information exchange with other organizations includes immunizations, laboratory and imaging reports, e-prescribing, and patient, administrative, and clinical decision support

Authorized staff and clinicians in participating health care organizations have access to patients’ information across providers and health systems

**Patient Portals (PPs) (** **16** **,** **18** **–** **20** **)**

Extension of an EHR that allows patients to access their health information and communicate with their provider’s team

Patients can ask questions, request prescription refills, schedule appointments, and obtain billing information

Information is controlled and managed by the health care organization or provider

Patients might require access to several PPs to gain access to all their health information if they see multiple providers across health systems

**Personal Health Records (** **16** **,** **19** **,** **20** **)**

Allows patients to gather, maintain, track, and control access to their health information in a secure and confidential environment

Contains information similar to an EHR and uses interoperability standards to pull information from other relevant sources

Patients can add their own information (eg, exercise and eating habits)

Patients can access information via a website or mobile application

At the county level, population health information is increasingly accessible to all stakeholders. Notably, the Robert Wood Johnson Foundation’s County Health Rankings and Roadmaps to Health program is providing county level tools, standardized information and measures, and guidance for improving population health (21). Their web-based County Health Rankings tool aggregates a broad array of health information for end-users to download, measure a county’s health, and make comparisons over time to other counties and the nation. This information drives health improvement decision-making and action at the county level. Roadmaps to Health provides guidance to aide multisector community organizations and members in working together to improve health (21).

Improving US population health requires a multisector and multiscale strategy (5,8). Health information systems are playing an increasingly significant role in addressing US population health for a range of end-users and geographic scales. What is missing is a record of health that facilitates multisector collaboration, information exchange, and integration at the community.

## Defining and Developing a Community Health Record

### Overview

We propose the community health record as a flexible model for how multisector community health stakeholders can use technology to aggregate and use information to better understand, address, and monitor their community’s health and its determinants. We define the community health record as both a framework to guide health care, public health, and community collaboration and information exchange and as a tool for integrating and transforming multisector data into information that can aid decision makers.

Informed by the EHR, personal health record, and the County Health Rankings and Roadmaps to Health systems, the community health record (framework and tool) will facilitate the exchange of relevant multisector information to its end-users to aid community health improvement. The principal difference is that the community health record integrates and presents multisector information at scales ranging from residential address to census block, census tract, neighborhood, or zip codes. Moreover, the community health record is communally owned and therefore requires a significant social component to initiate and sustain collaboration and information exchange between stakeholders. The goal is to inform, target, monitor, and evaluate a portfolio of evidence-based community health interventions, recognizing that community health issues can be simultaneously addressed across the spectrum of health by multiple community health stakeholders from various sectors (5,22). Collectively, these efforts provide a foundation for health care, public health, and community partners to better understand and manage the health of their populations.

In Shelby County, Tennessee, the Shelby County Health Department, Methodist Le Bonheur Healthcare, the Common Table Health Alliance, Tennessee Department of Health, state of Tennessee, and the Centers for Disease Control and Prevention (CDC) are piloting community health record development for heart disease and stroke. We use examples from the Tennessee pilot to illustrate the community health record framework in practice.

### Framework

**Figure 1:**
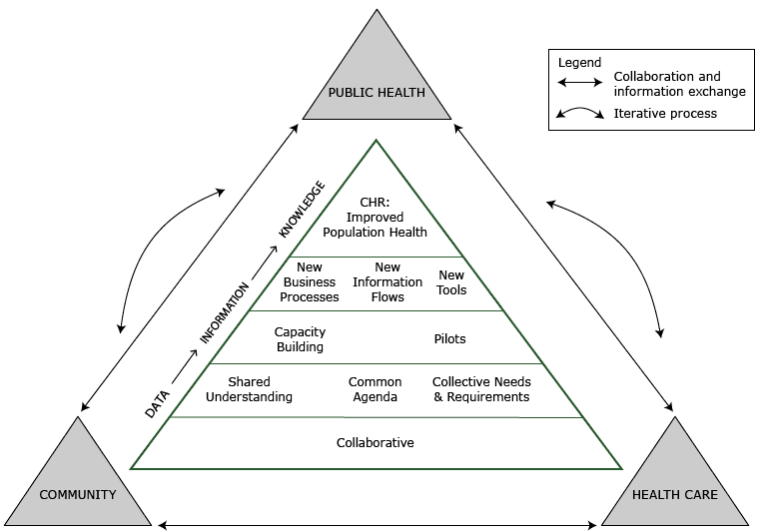
The community health record framework. The framework presents a multitiered, multisector model illustrating an iterative, flexible, and participatory process for achieving collaboration and information exchange among health care, public health, and community groups and organizations to aid population health decision making. Abbreviations: CHR, community health record; CH, community health.

The community health record framework (Figure 1) is a multitiered, multisector model proposed to facilitate the development of a community health record. It describes an iterative and participatory process for achieving collaboration and information exchange between health care, public health, and community organizations. The aim is to 1) enable meaningful collaboration, 2) facilitate a shared approach, 3) build workforce and infrastructure capacity, and 4) establish a new way of doing business that enables the transformation of community health data into information and information into knowledge to aid decision makers in collectively improving population health. The framework identifies concepts necessary for each aim and proposes an infrastructure to facilitate community health record development.

#### Aim 1. Collaboration

**Collaborative.** The foundation of the community health record is establishing a high-functioning collaborative of engaged community health stakeholders. Real collaboration occurs when stakeholders agree to align efforts and share information to address a common goal (23). These collaborations require trust and time to develop and begin with focusing on a win–win outcome. They rely on a core group of project champions from each organization who are willing to listen, share responsibilities, do things differently, pool resources, work together, and take collective action (6,23). Sustaining the collaborative is vital for information sharing (24). Success is achieved when the collaborative collectively invests in a new way of doing business (23,25). In the Tennessee pilot, stakeholders recognized the need to come together and develop a new way of doing business to collectively use data from each of their organizations to improve health.

#### Aim 2. Shared approach

**Shared understanding.** Collaboratives that achieve collective impact (25) are characterized by stakeholders who work collectively to develop a shared understanding of their problems and potential solutions and a common agenda for resolving (25). Therefore, the next step in the community health record framework is to develop a shared understanding of the problems and proposed solutions. This requires that stakeholders understand each of the problems and potential solutions from their own perspective and the perspective of their collaborators. It includes understanding each stakeholder’s value propositions. To achieve this understanding, collaborators should collectively define an overarching set of project objectives as well as subobjectives for each stakeholder. The subobjectives are those objectives that each stakeholder needs to accomplish for the project to have value for their organization (23). For example, the primary objective for all community health record stakeholders in the Tennessee pilot is to use the community health record to facilitate chronic disease information exchange and health improvement. For Methodist Le Bonheur Healthcare, the subobjective is to use the community health record for the required Internal Revenue Service Community Health Needs Assessments. The Shelby County Health Department’s subobjective is to use the community health record for subcounty chronic disease surveillance. Collectively focusing on both objectives and subobjectives ensures a degree of success for all.

**Common agenda.** Achieving these multisector objectives is a substantial undertaking that requires thoughtful development of a common agenda and the processes and practices necessary to outline the roles, responsibilities, tasks, and deliverables to execute, monitor, and evaluate the project. The best practices and tools of project management can ensure a thoughtful and collective approach to achieving success and avoid unnecessary misunderstandings and conflicts (26). To develop a common agenda in the Tennessee pilot, a stakeholder significance analysis was conducted to facilitate a common understanding of the interest, influence, risk, and reward of participating stakeholders (Appendix A). In addition, stakeholders collectively developed a project charter and schedule to define and document the scope, objectives, roles, responsibilities, milestones, timeline, and communication strategy (for guidance and templates, see http://www2a.cdc.gov/cdcup/library/templates/default.htm).

**Information needs and requirements.** Having reached consensus on the shared problems, solutions and an agenda, the next step is to collectively define relevant measures to assess community health determinants and outcomes. Community health stakeholders need an array of clinical, public health, socioeconomic, environmental, and behavioral data and measures at relevant temporal and geographic scales to understand health, document disparities, and design and target effective interventions (6). It is important to identify and prioritize measures that are operationally feasible and balanced across stakeholder needs, focusing on the minimal set necessary to aid decision making and prevent unnecessarily burdening data providers (27). Collectively adopting a core set of standardized measures at defined spatial and temporal scales is useful in enabling community groups to monitor, compare, and evaluate community health interventions by time and geography. Selected measures should be mapped to specific databases to ensure access and availability of data at the required spatial and temporal scales. Tennessee pilot stakeholders initially identified a wish list of more than 100 measures (health outcomes and determinants). Through a process of prioritization, mapping, and clarification of purpose, the list was reduced to fewer than 20. Information access and availability will also be determined by data use agreements between information providers and end-users. The end result is a compromise among need, privacy, security, confidentiality, and trust.

Using the diverse and increasingly vast amount of community health data requires an information system for exchanging and integrating community health data to make it useful to end-users (6). The utility of such a community health record tool should be maximized by defining stakeholder requirements (28). Individual and collective stakeholder use cases and scenarios are useful methods for capturing data on requirements and information needs (29). Requirements were identified in the Tennessee pilot through small group discussions, stakeholder scenarios (Appendix B), and user-interface mockups.

#### Aim 3. Workforce and infrastructure capacity

**Capacity building.** Capacity building is the most important step in the community health record framework. Developing, implementing and sustaining the community health record requires that multisector stakeholders develop the necessary epidemiology, informatics, and technical expertise and resources. Capacity building is essential in empowering communities to take ownership, partner, and achieve self-sufficiency. It could include chronic disease small-area epidemiology and informatics methods and workforce development, health information technology infrastructure and resources, and community-based trainings (30–32). Capacity building should occur throughout community health record development and use, but it is particularly relevant at the pilot projects phase because it provides the opportunity to assess and initiate the expansion of local capacity before a significant investment in a new process and system. For example, to expand public health informatics capacity in Shelby County, staff members from the Shelby County Health Department and Methodist Le Bonheur Healthcare piloted the local CDC Informatics Training in Place Program (I-TIPP). The training environment was essential in facilitating the initial exchange and analysis of health care and public health information.

**Pilot projects.** Pilot projects are a critical component of the community health record framework. They enable stakeholders to explore the processes required and the value of the data, identify problems, and design, implement, and evaluate solutions. Small wins often enable stakeholders to garner support and trust from their leadership and collaborators to undertake more prominent future efforts (27). For example, Tennessee I-TIPP success spawned additional projects investigating the epidemiology of hospital readmissions and usefulness of EHR data for population health. Pilot projects also provide the opportunity to assess the current conditions and explore the epidemiology and informatics methods, human and technical infrastructure, data access, and automation needed for sustainability. These projects give the collaborative the opportunity to learn by doing and to identify unforeseen issues, needs, and opportunities before scaling. In the Tennessee project, stakeholders piloted the use of subcounty vital statistics and discharge data for chronic disease surveillance. Community leaders expressed interest in receiving information at this scale. The Shelby County Health Department realized, however, that they could not sustain such an effort without additional staff or automation.

#### Aim 4. A new business model

**Business processes.** Business process analysis and redesign methodology (33) allows stakeholders to describe their current business processes (“the way in which organizations conduct their activities and achieve specific goals and objectives” [33]) and propose new processes to support collaboration, success, and sustainability. By characterizing their existing and proposed processes, stakeholders identify their business goals, objectives, triggers, inputs, outputs, rules, and outcomes (33). Outcomes and lessons learned from the previous steps will inform and refine the new processes. Stakeholders may have to adapt their business processes to work across sectors with multiple stakeholders in the evolving population health environment. For example, health care organizations are engaged in population health management, and local public health is working with health care to address chronic diseases. This requires local public health to shift from a largely communicable disease focus to one that places equal importance on chronic disease (31) and requires health care to move outside the walls of the hospital (34).

**Information flows.** Efficient and timely access to relevant multisector community health information depends on interoperability, the ability of different systems and organizations to easily exchange and use information (35). While technical interoperability is important, the difficulty lies in resolving the complex social and organizational dynamics associated with interoperability (24,36). It is about getting multisector organizations to exchange information within an information technology environment (35). The initial steps in the community health record framework illustrate that this process is largely social. Long-term sustainability requires leadership, institutional change, trust building, collaboration, legislation and policy, and resources as well as standards and technology. To resolve these complex social issues in the Tennessee pilot, it was necessary for stakeholders to agree that data providers would govern access to their information and recognize that different stakeholders would have different levels of information access.

Although the end goal is the development of shared information systems for provision of community health information, communities should initially focus on iteratively advancing their current capacity by achieving small wins, building trust, and securing resources. For example, clinical and public health stakeholders in Shelby County were not sharing clinical information. It was therefore appropriate to start with secure electronic data exchange and collective analysis and interpretation before moving to an automated system. Incrementally moving from the simple to more technically advanced states still requires resolving many complex social issues as well as data format, standards, and quality issues. Regardless, sharing data should begin with establishing a data use agreement to ensure that privacy, confidentiality, and security issues are addressed (37) (for guidance and a template, see http://www.hsrmethods.org/PrivacyInResearch/Privacy%20Tools/Guidance%20on%20HIPAA%20Data%20Use%20Agreements.aspx).

**Figure 2:**
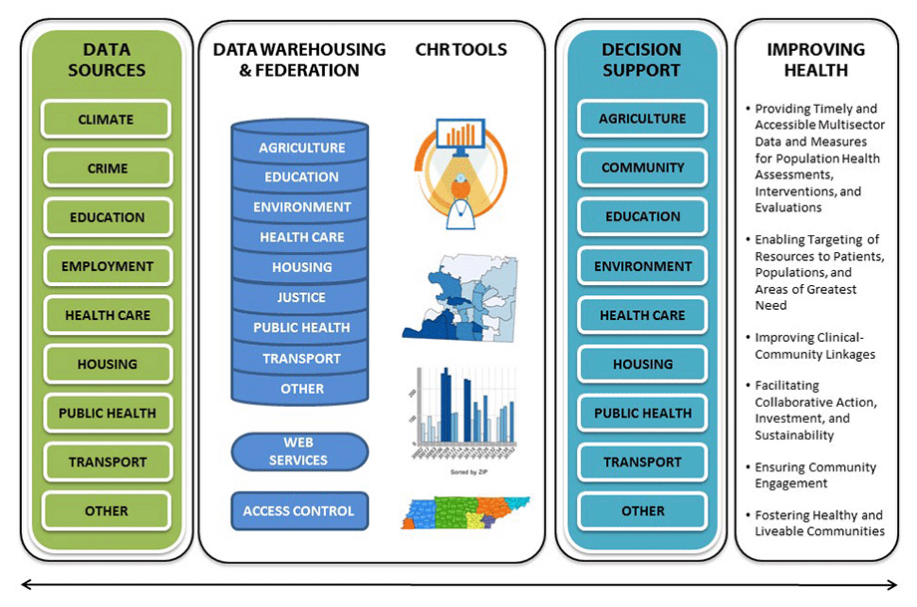
Conceptual model of the multisector community health record (CHR) tool. The underlying infrastructure consists of open-source software, services, and tools that leverage open standards. The model is illustrative of 1) the multisector data sources, 2) the implementation of a secure federated data store and warehouse with complementary web services, and 3) tools for providing multisector end-users with information to collectively improve health outcomes.

**Tools. **Achieving and sustaining the community health record vision requires the development of a dynamic information technology infrastructure that satisfies the needs of multiple sectors (Figure 2). The community health record tool should enable the integration, analysis, and visualization of information from multiple sources. Its common infrastructure must be standards-based and promote the synthesis of data management, data policy, and information systems solutions by leveraging open and well-established standards (eg, Health Level Seven [HL7] [http://www.hl7.org/implement/standards/]) to the extent possible. The use of an open platform that is designed to be repeatable, adaptable, scalable, and nodal will allow a natural network effect. This will facilitate the automation of the collaborative exchange of information and accelerate the discovery of linkages between practices, context, and data. Pilot projects should validate the feasibility and utility of open-source tools and open-data standards including evolving industry-standard data stores and warehouses, a federated data gateway to support sharing of emerging data stores, and an analytics and visualization infrastructure in various community health environments. The prototype community health record architecture and user interface are presented in Appendix C and Appendix D, respectively.

## Conclusion

Improving community health begins with real collaboration, access to relevant information, and development of appropriate infrastructure and tools for community health stakeholders to effectively manage and use information to aid decision making. The goal of the community health record framework is to help facilitate sustainable collaboration, information exchange, and collective action among community health stakeholders to address shared goals and improve health outcomes. The framework illustrates an iterative and participatory process for achieving this goal. It is meant to be flexible. Through the community health record framework, community health stakeholders have an opportunity to collectively address health within a community by bringing people, organizations, information, and technology together to document measures of health and determinants, identify needs and priorities, and target and evaluate interventions.

Historically, data sharing projects have been ad hoc, inconsistent, and limited in scope. The lack of multisector collaboration, shared tools and data infrastructure, and governance; fragmented policy; and limited resources are core barriers to realizing the inherent promise of integrated information exchange. The changing population health environment represents an opportunity to leap-frog many challenges and realize current solutions and future advancements in multisector collaboration, information exchange, and technology. The community health record provides a framework and tool to overcome these barriers and achieve such a solution.

## Appendices

### Appendix A. Stakeholder Significance Analysis of State and Local Community Health Record Stakeholders at Project Outset.

This appendix is available for download as a Microsoft Word document.

### Appendix B. Example of User Scenarios Developed by Tennessee Community Health Record Project Stakeholders.

This appendix is available for download as a Microsoft Word document.

### Appendix C. State of Tennessee Community Health Record (CHR) Infrastructure.

This appendix is available for download as a Microsoft Word document.

### Appendix D. Selected Screenshots of the Prototype Community Health Record User Interface Developed by Weave Visual Analytics, With Local and State Community Health Stakeholders in Shelby County, Tennessee.

This appendix is available for download as a Microsoft Word document.
