# Supplementary material for: A Community Health Record: Improving Health Through Multisector Collaboration, Information Sharing, and Technology
Source: Prev Chronic Dis. 2016 Sep 8;13:E122. doi: 10.5888/pcd13.160101 (PMC5027852; doi:10.5888/pcd13.160101)
Supplement: Supplementary file 1 [file 16_0101_AppendixA.docx]

**Appendix A:** **Stakeholder significance analysis of state and local Community Health Record stakeholders at project outset.**

The results of the stakeholder significance analysis^1^ illustrate the significance of each stakeholder in achieving project goals. In particular, they highlight Methodist Le Bonheur Healthcare (MLH) as the definitive stakeholder due to the risk associated with the secondary use of electronic health record data for population health. As a result, all stakeholders and project decisions seek to minimize this risk to MLH and ensure patient privacy and confidentially is of utmost importance.

|  | **Stakeholder Attribute** | | | |  |
| --- | --- | --- | --- | --- | --- |
| **Stakeholder Type** | **Interest** | **Influence** | **Risk** | **Reward** | **Stakeholder** |
| **Discretionary** | Yes | No | No | No | Other Non-profit |
| *Interest but no power to influence or directly benefit* |  |  |  |  |  |
| **Dormant** | No | Yes | Yes or No | No | Other local government |
| *Potential to have a direct impact on the project but lacks interest, reward and/or risk is too great* |  |  |  |  |  |
| **Dependent** | Yes | No | No | Yes | Common Table Health Alliance |
| *Strong interest in the project, stand to reap benefits but unable to act on own interest* |  |  |  |  |  |
| **Dominant** | Yes | Yes | No | Yes | Shelby County Health Department; TN Department of Health |
| *Strong interest in the project, have power to influence its direction, stand to reap benefits but have little risk* |  |  |  |  |  |
| **Definitive** | Yes | Yes | Yes | Yes | Methodist Le Bonheur Healthcare |
| *Key player with strong interest and influence that stands to reap benefits but is exposing themselves to potential risk* |  |  |  |  |  |

^1^ Adapted from Bunn MD, GT Savage, and BB Holloway. (2002) Stakeholder analysis for multi-sector innovations. The Journal of Business & Industrial Marketing 17(2/3): 181-203
