# Supplementary material for: A Community Health Record: Improving Health Through Multisector Collaboration, Information Sharing, and Technology
Source: Prev Chronic Dis. 2016 Sep 8;13:E122. doi: 10.5888/pcd13.160101 (PMC5027852; doi:10.5888/pcd13.160101)

**Appendix B:** **Example user scenarios developed by TN Community Health Record project stakeholders.**

**Methodist Le Bonheur Healthcare (MLH)**

**User Scenario 1: From the perspective of the Chief Financial Officer at MLH, wanting to focus on prevention and improving overall health status of the community at large, as well as protecting our margin.**

Fast forward to 2014, when the readmission penalties are at full force. You are the CFO of a large, multi-hospital health system, with many hospitals in the urban core of a city of people with multiple co-morbidities, poverty, and overall poor health status, including obesity and cardiovascular disease (particularly congestive heart failure). Your health system provides more than its share of care for the indigent and under-insured population and the majority of your patients have Medicare as a payer. You are at risk, especially in light of the non-payment for readmissions that has already started and will increase over the next two years. You have “gone lean” in all the ways you can in terms of the operating of both your inpatient and ambulatory care sites and have acquired numerous primary care practices in the past 18 months, with a huge outlay of capital, to try to position your system for the future of bundled reimbursement. You need solutions from outside the hospital and community alignment to maintain financial solvency with these new reimbursement structures.

You get a call from your new CEO, asking how we are doing about preventing readmissions in zip code A, one of “hotspots” in terms of acuity of illness, high levels of readmissions and high amounts of write off or charity care. Due to the creative development of the CHR in 2012-2013, you can quickly go to the CHR dashboard and pull up the last quarter’s stats on not only our readmission, charity write off and acuity of illness in that zip code, but also, what percentage of persons there have a regular primary care home, what percentage come in to the hospital via the ED or through ambulatory care offices, as well as what percent of net income is spent on healthcare per household. You log in, select the Access column on the dashboard and then zoom in to zip code A. You are pleased to see, thanks to the effort of this group, that MLH has greatly reduced readmissions and can quickly calculate for your CEO what that means in dollar savings to the system, as well as avoiding further penalties going forward.

Additionally, you see some other metrics there that make you feel more confident about keeping the hospital bottom line more robust, that relates to prevention efforts. On this dashboard, you can see the number of people in the community who are getting regular wellness visits, charted by age, as well as the number of people trained in disease self-management through the Congregational Health Network (CHN) a 500+ church and hospital partnership designed to improve access and health status of all. Approximately 10 local churches have been strong partners in that zip and surrounding areas since 2006 and have worked intentionally with MLH to offer mobile clinical services and navigation to local safety net clinics on a monthly basis since Jan. 2013. Lastly, you scroll through to quickly ascertain the % of those graduating from high school and those employed, which are social determinant proxies for improved health status in the future. You are pleased to see big changes in those social determinants, with more persons employed and completing a HS equivalency. Also, you see an increase in home ownership, always a good sign for a neighborhood, in terms of rebound and growth AND ability to get insurance coverage and/or pay healthcare expenses.

**User Scenario 2: From the perspective of a Congregational Health Network Partner, wishing to improve their church’s neighborhood, help with housing and economic and workforce development, as well as education and health status of zip code A residents.**

It’s 2014 and you are a dedicated, 65 year old pastor, with a well-established health ministry in zip code A. You and your parishioners and health ministry staff have been working in zip code A for over 15 years, to improve the health status and overall quality of life, admittedly a “tough” neighborhood, with higher than usual incidence of violence, poverty, sub-standard housing, blight and environmental toxins. You have been working with local, state, and federal community health partners to create a Community Health Record (CHR) that is meaningful and useful to you and the local residents, not just to hospital or public health or other stakeholders. Through a series of participatory conversations, you and your staffers and local residents helped create a record that answers your questions and really helps you and the residents in improving quality of life and health status.

You get a call from the city Office of Faith Based Initiatives, who need to know how many of your local residents have had an annual wellness exam (as they are applying for Federal funding) and you quickly log in to the CHR, select access and zoom to zip code A, and further to neighborhood B and give them an answer on prevalence. You are so pleased to see that percent rise over 10% since this metric was first monitored in 2013 and take that as a good sign that MLH staff are doing a better job of navigating residents to appropriate safety net clinics and other mobile clinical services, for improved continuity of care and follow-up. The CHN training in navigating the health system has also “demystified the process” for local residents and increased their level of trust in the hospital, local clinics and their providers. You also can go to the CHR and pull out how many of zip code A residents are getting preventive services, like mammograms, as this area was one of the areas in Memphis or any other city with the highest percentage of breast cancer mortalities for African-American women in 2010, which you can see has changed significantly for the better now in 2014. Happily, you note that over 500 people have been trained in self-management of disease through the Congregational Health Network (CHN) training administered in this zip code.

While you are pleased with these improvements in health status, you are pleased that MLH, Shelby County Health Department, CDC and other partners are planning in 2015 to “cross-walk” or add other indicators to this record, like percent of blighted property deeded to clergy or others for community gardens and percent of acreage containing organizations which have been fined for high levels of toxic waste, which is another metric that reflects the improving health of your beloved Memphis and local community. With the addition of even more proxy measures for overall quality of life in zip code A, accessible to all via the open source and publically accessed CHR, everyone in Memphis is part of the solution to improve our city and our life and well-being.

**Shelby County Health Department**

**User Scenario:**
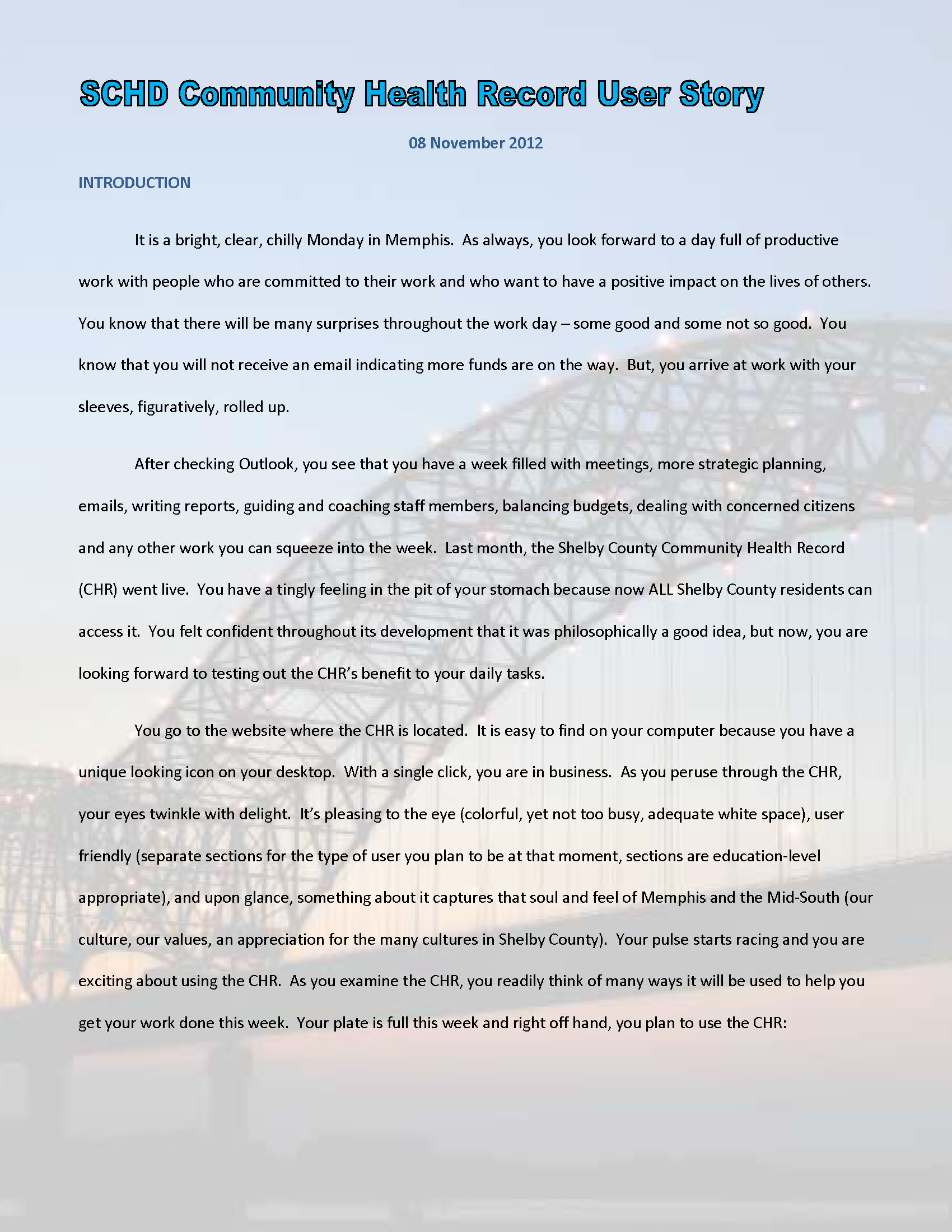


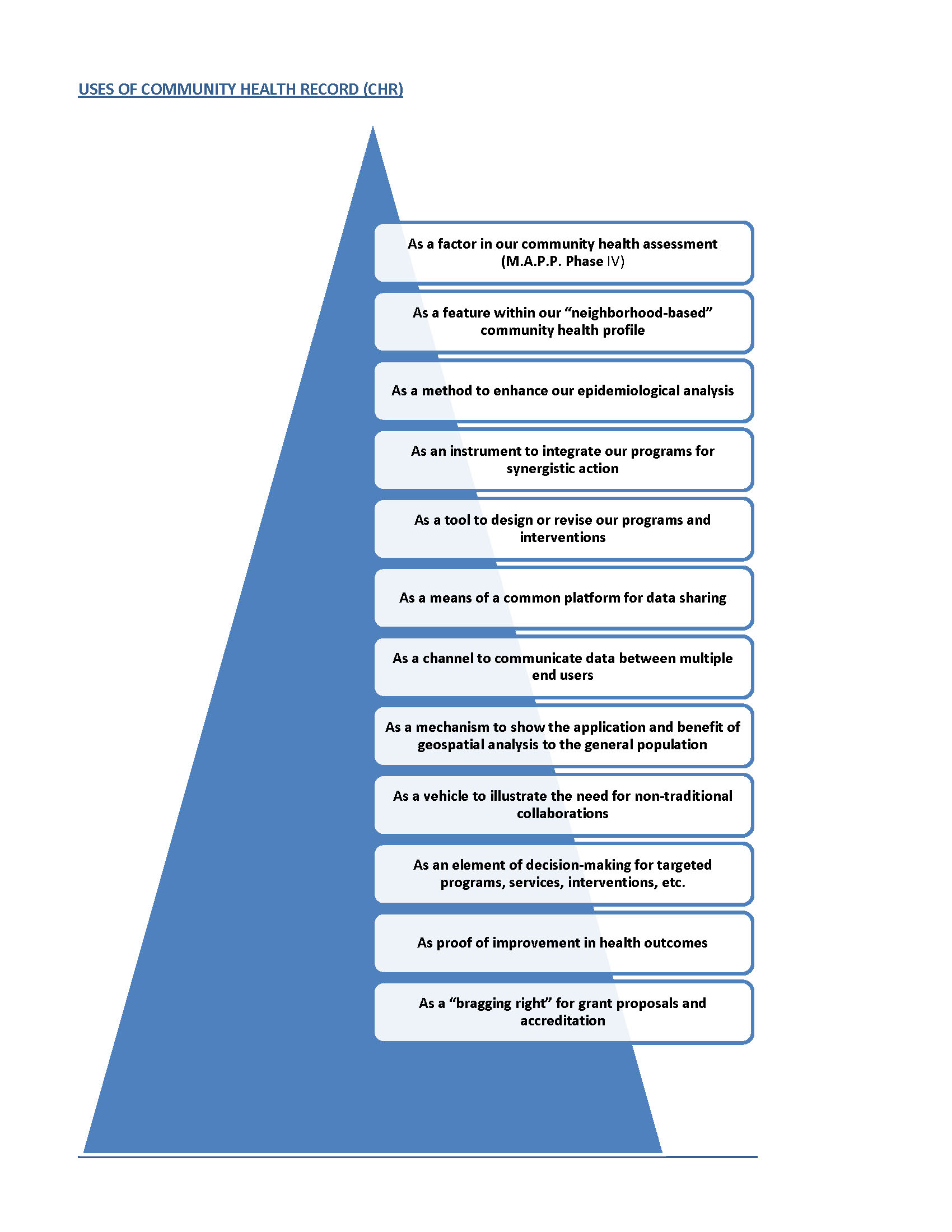


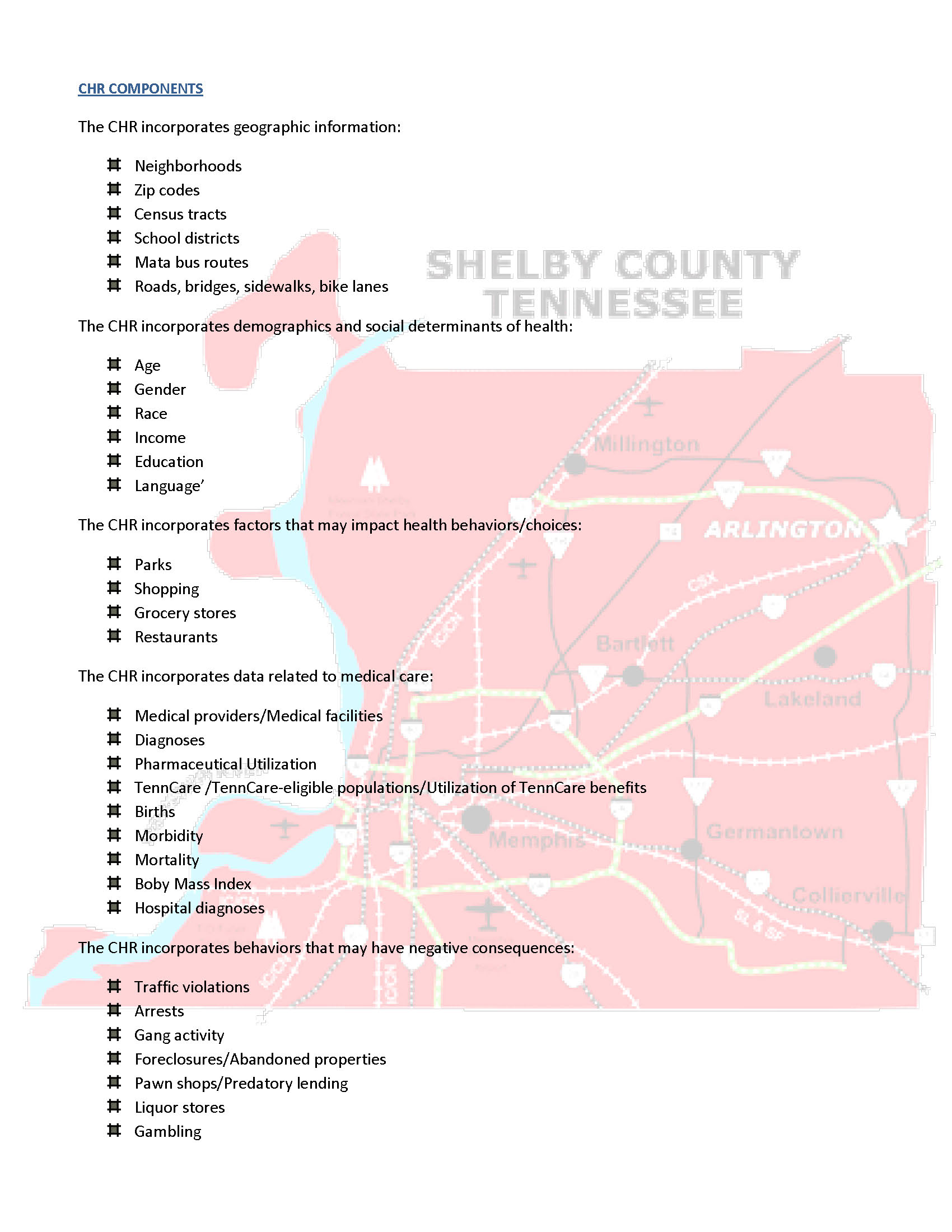


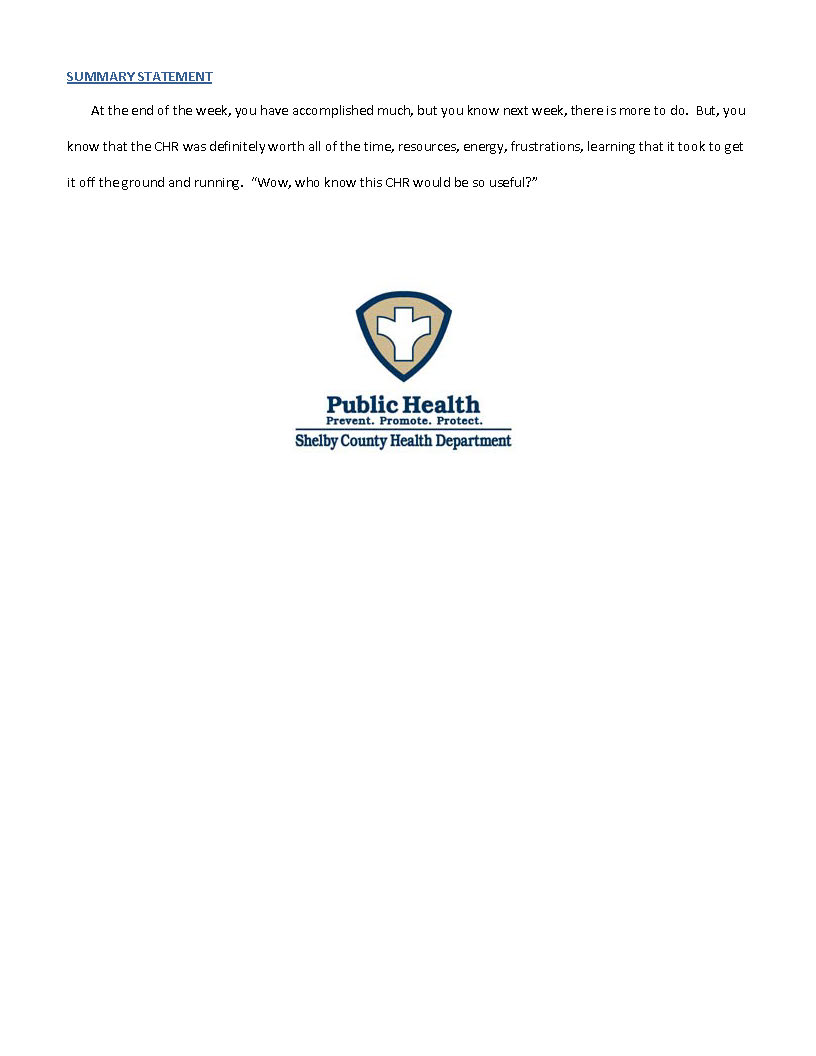

Supplement: Supplementary file 2 [file 16_0101_AppendixB.docx]
