# Supplementary material for: A Community Health Record: Improving Health Through Multisector Collaboration, Information Sharing, and Technology
Source: Prev Chronic Dis. 2016 Sep 8;13:E122. doi: 10.5888/pcd13.160101 (PMC5027852; doi:10.5888/pcd13.160101)
Supplement: Supplementary file 3 [file 16_0101_AppendixC.docx]

**Appendix C. State of TN Community Health Record (CHR) Infrastructure.** CHR tool infrastructure, utilizing Open Source and Open Data standards via the construction of an Open infrastructure. It includes 1) the implementation of an evolving industry standard data store (*mongoDB*) and warehouse (*Hadoop*); 2) the implementation of a federated data gateway utilizing *CKAN*, a nodal framework supports simplified cross-pollination of emerging data stores; and 3) the use of WEAVE ANALYTICS AVR infrastructure, a next-generation Business Intelligence (BI) platform supporting the construction of task-specific analytic views of the federated data <http://iweave.com/>.


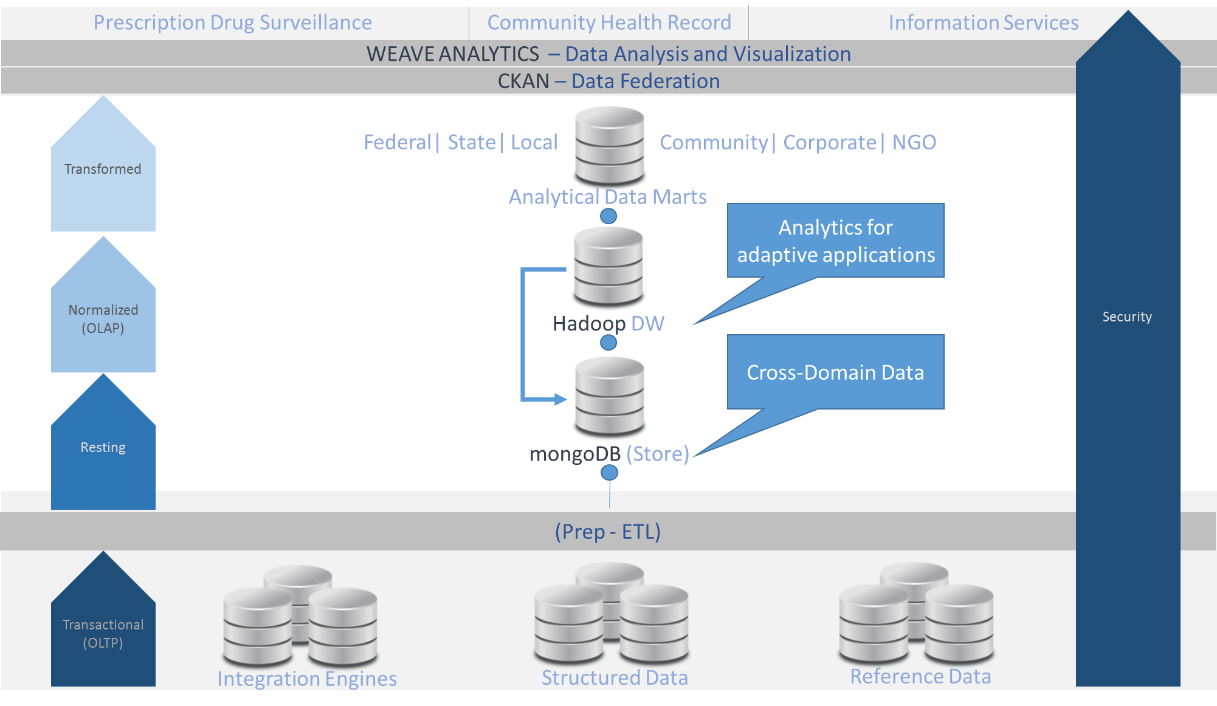


*The implementation is contingent on existing technical infrastructures, and may be substituted with like solutions as both the technologies and framework evolve.
