# Supplementary material for: A Community Health Record: Improving Health Through Multisector Collaboration, Information Sharing, and Technology
Source: Prev Chronic Dis. 2016 Sep 8;13:E122. doi: 10.5888/pcd13.160101 (PMC5027852; doi:10.5888/pcd13.160101)
Supplement: Supplementary file 4 [file 16_0101_AppendixD.docx]

**Appendix D:** **Select screenshots of the prototype Community Health Record user interface developed by Weave Visual Analytics,** <http://iweave.com/>, **with local and state community health stakeholders in Shelby County, TN.**

All data featured is synthetic and for conceptual purposes only.


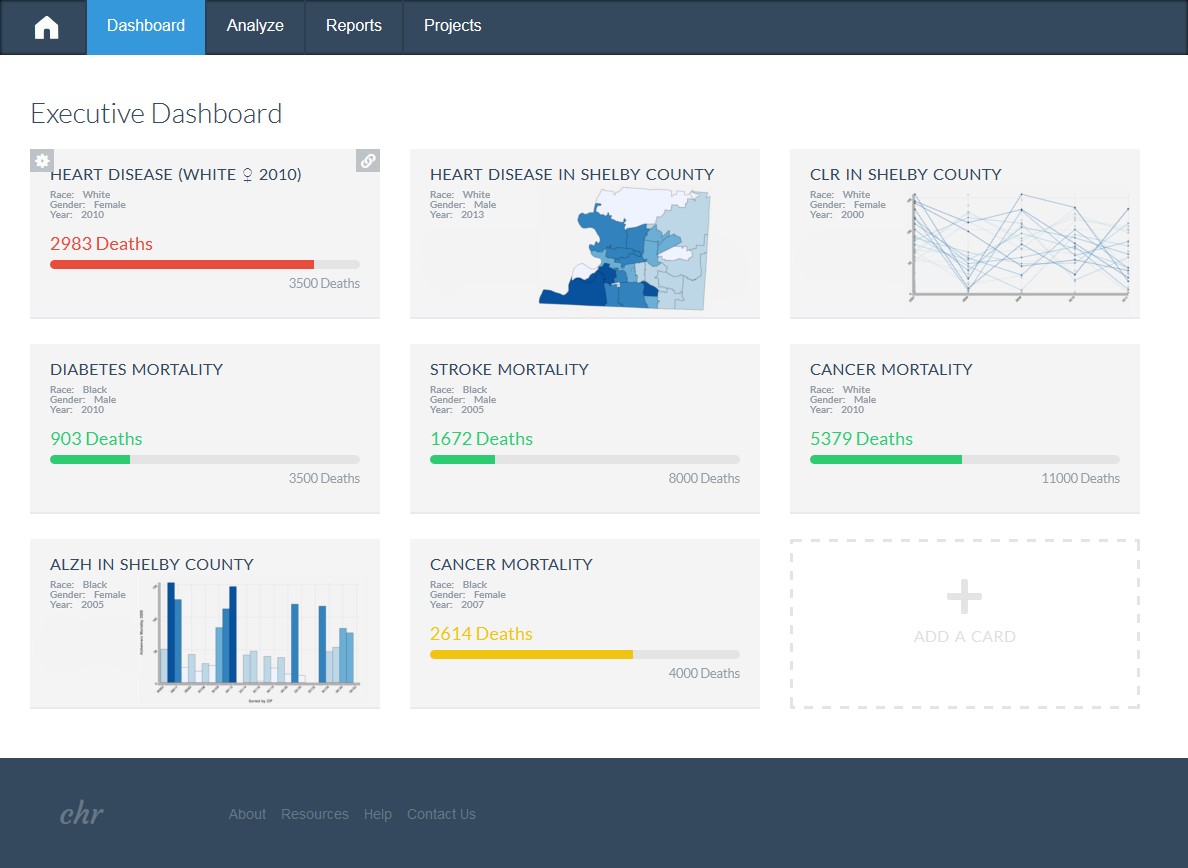


**Figure 1.** Community Health Record landing page – Executive Dashboard. Its card-based design provides a flexible and quick update of the information that the end-user has selected as most important to track.


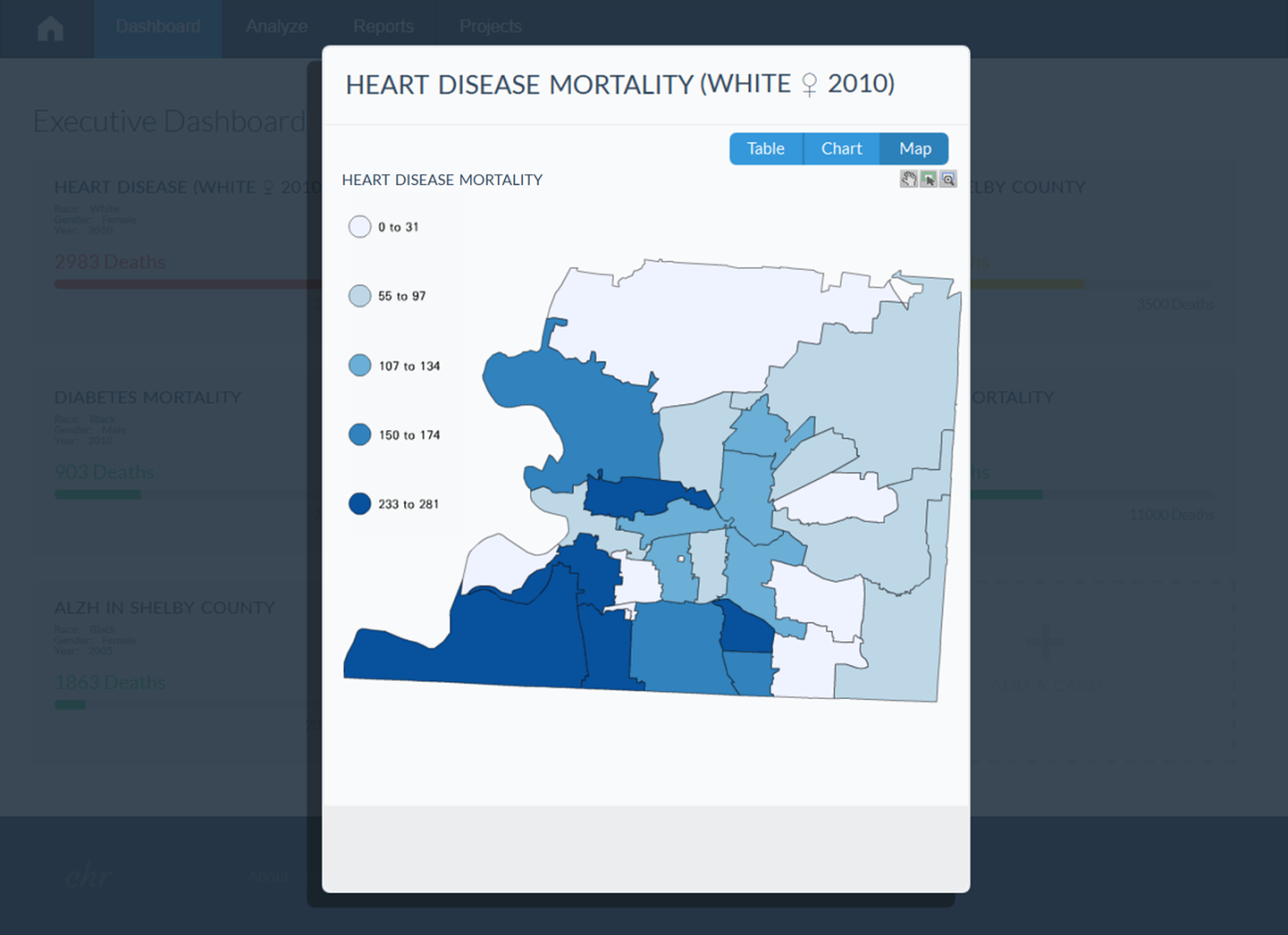


**Figure 2.** The Community Health Record Quick-View Modal provides a more detailed view of the information on the Executive Dashboard cards in table, chart or map views.


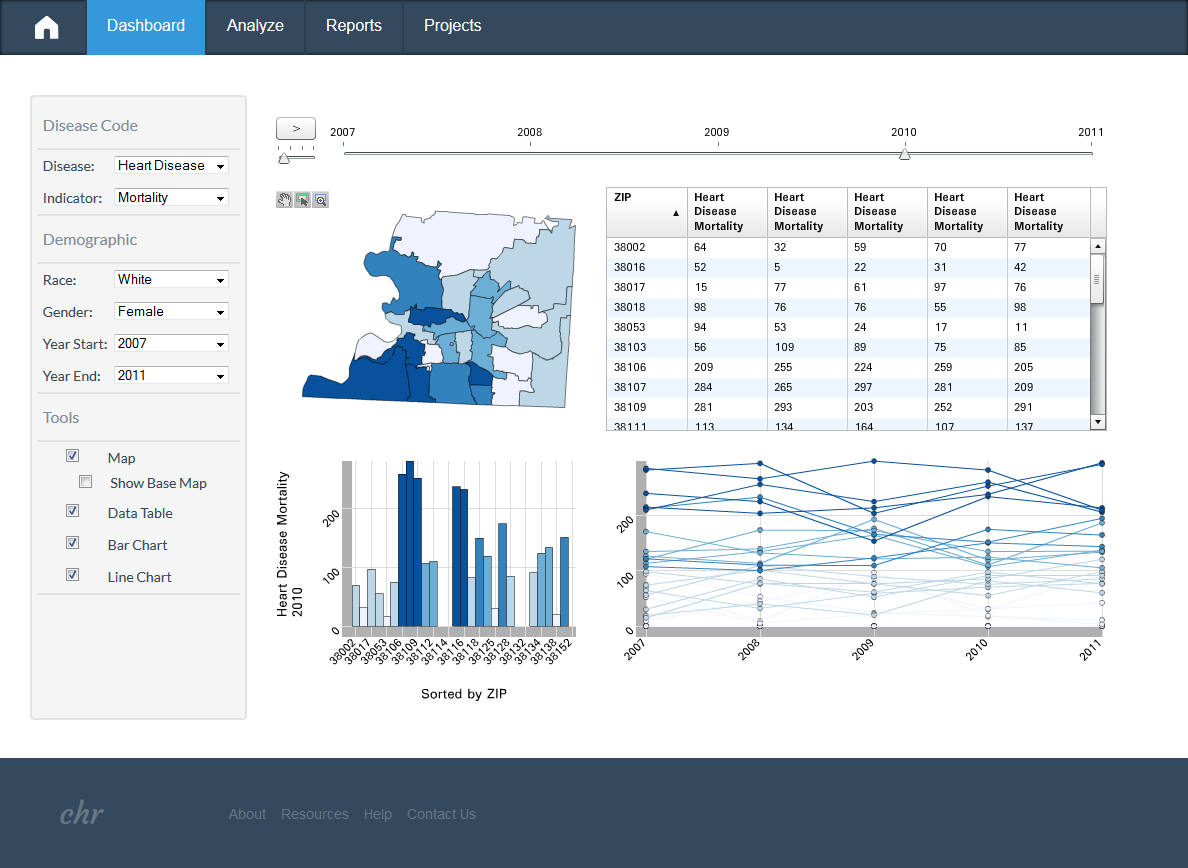


**Figure 3.** Community Health Record Epidemiology Dashboard allows end-users to quickly select, filter and visualize information in their database in map, table and/or chart views. The timeline function at the top of the dashboard allows the information selected to be visualized temporally.


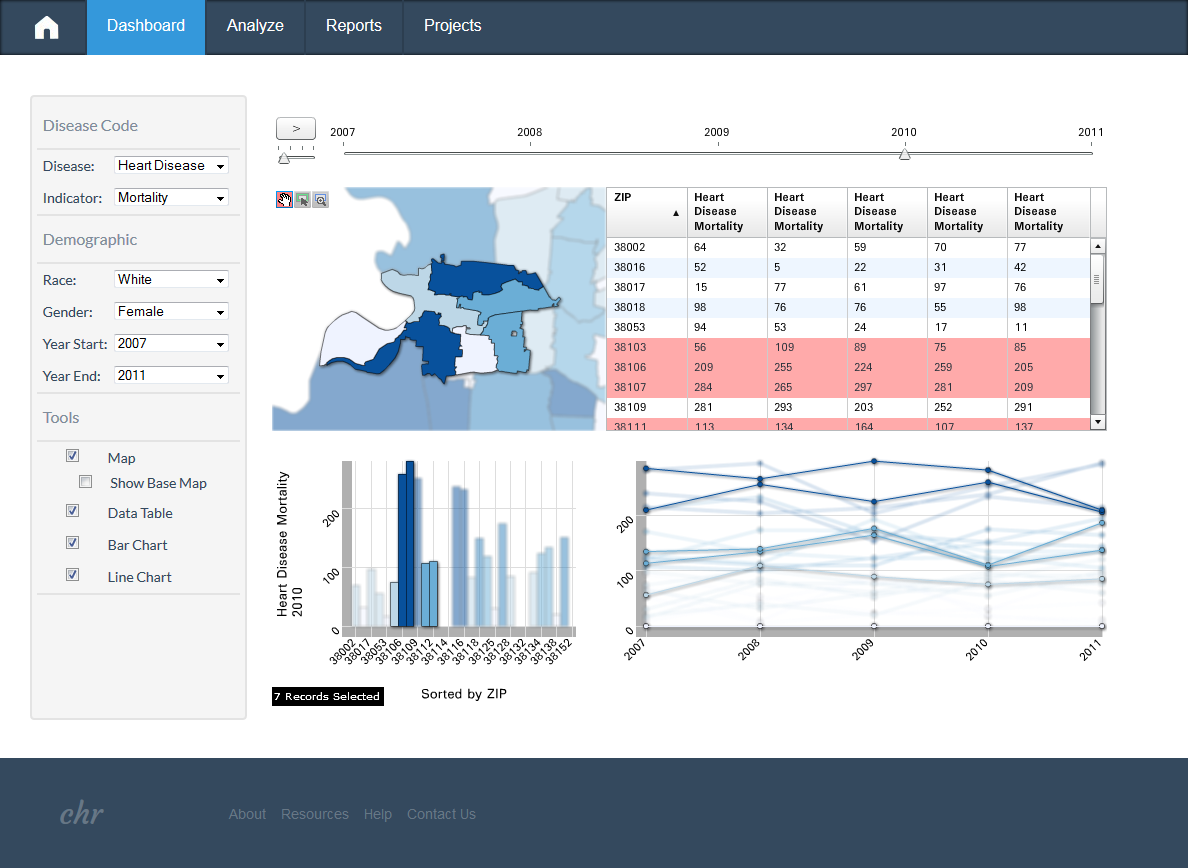


**Figure 4.** The end-user may subset select areas and attributes on the Epidemiology Dashboard by selecting areas or information of interest on the visualizations. A right-click over a visualization or table allows all or a subset of information to be tracked on an Executive Dashboard card, exported for analysis or printed.


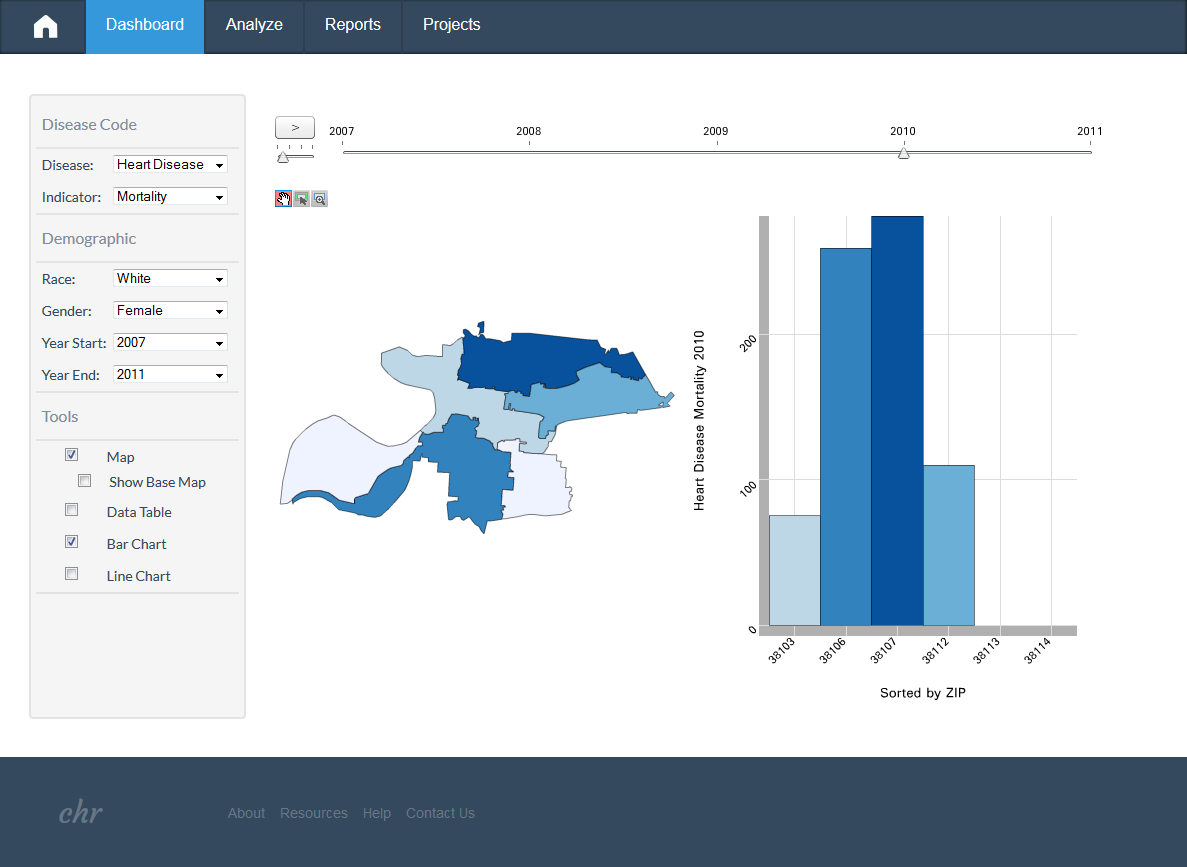


**Figure 5.** The Epidemiology Dashboard allows the end-users to turn visualization tools off and on as needed.


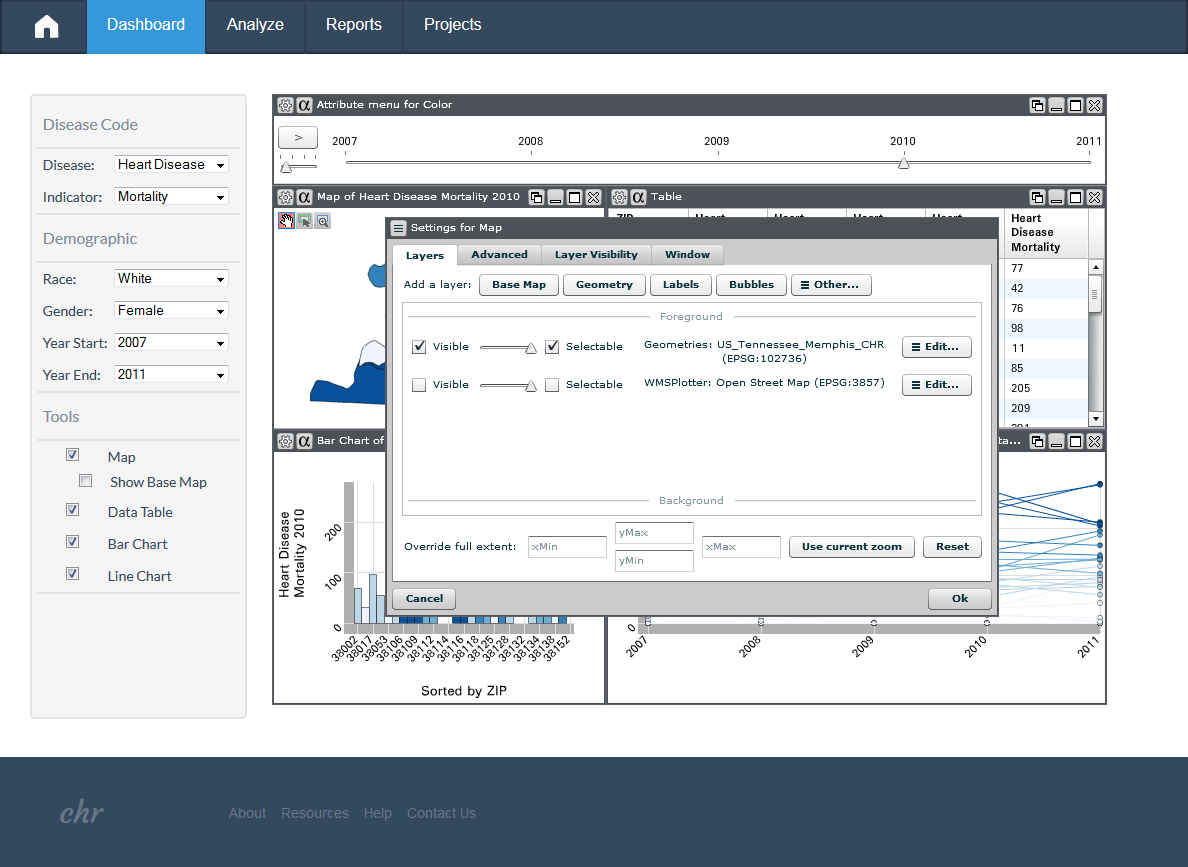


**Figure 6.** The Community Health Record Edit-Mode allows the end-user to edit the Epidemiology Dashboard. Users can change the appearance and location of any visualizations, e.g. data layers, colors, titles, etc. in a Weave session, <http://iweave.com/>.


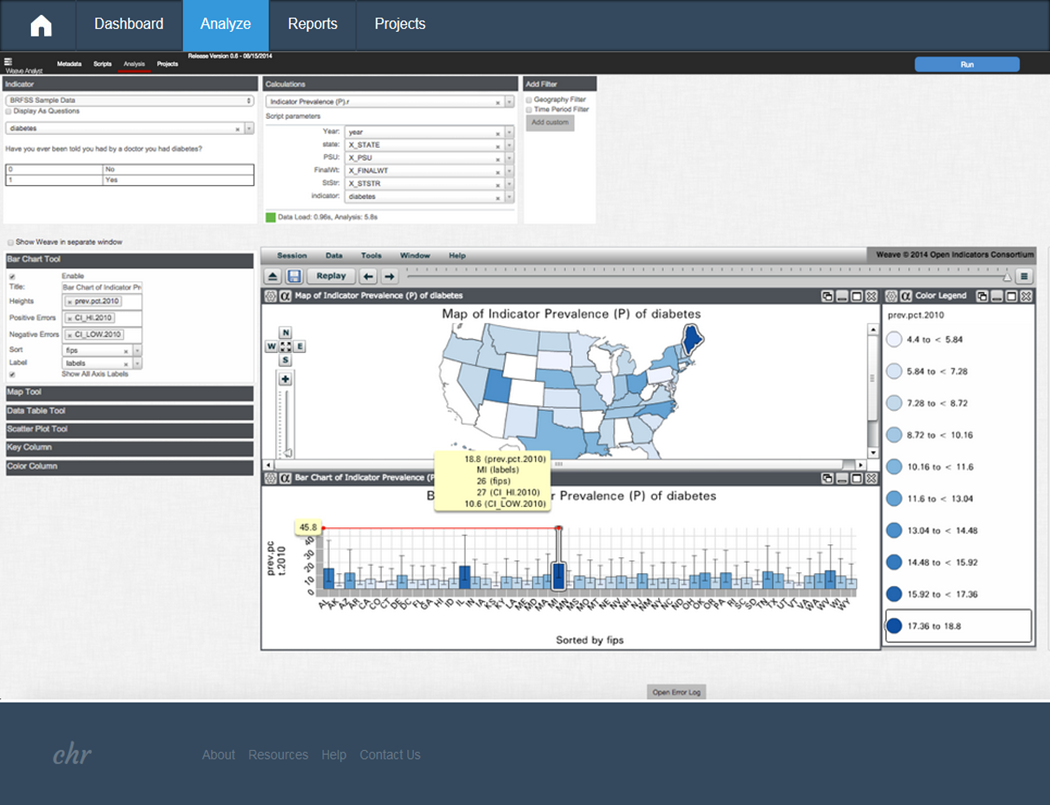


**Figure 7.** The Community Health Record Analyze tool allows end-users to analyze their data for visualization or other purposes using Weave Analyst. Weave Analyst is currently in deveolopment, <http://iweave.com/>.


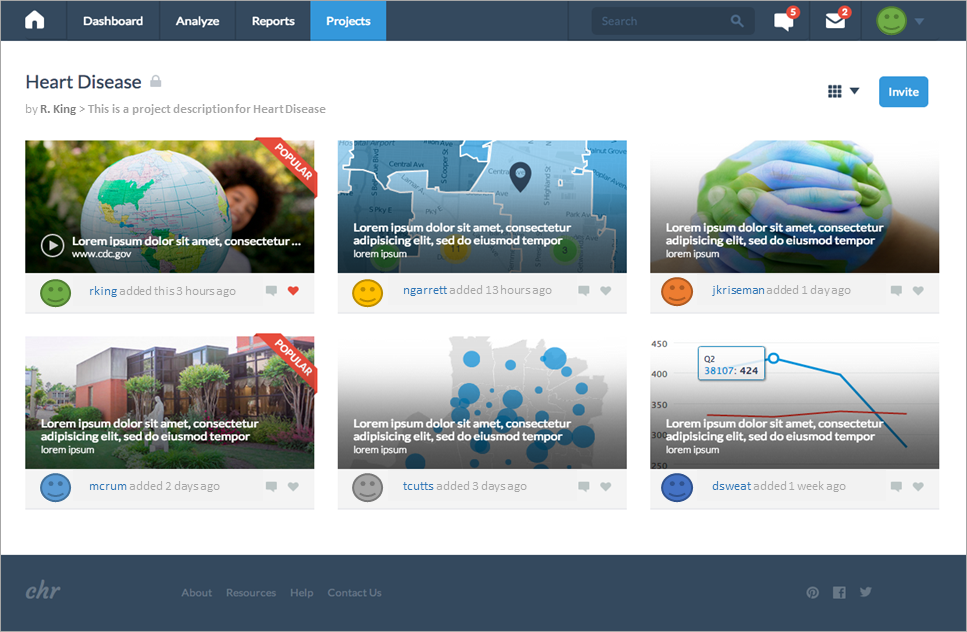


**Figure 8.** The Community Health Record (CHR) Projects tool allows end-users within and across organizations to organize and virtually collaborate on projects within a secure environment.


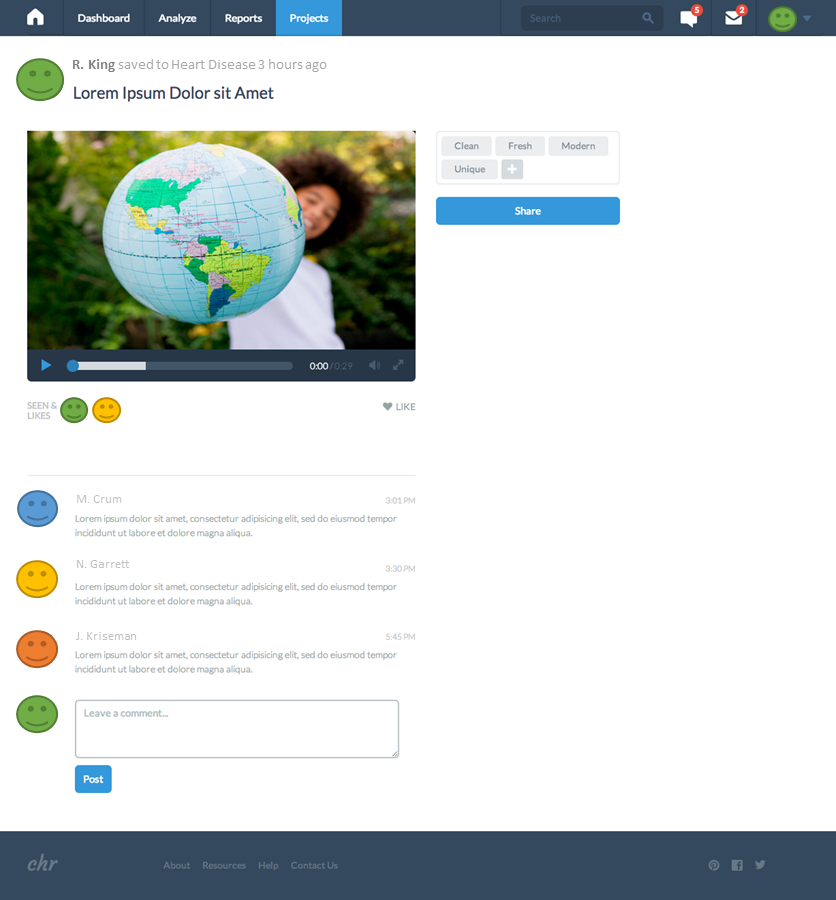


**Figure 9.** The Community Health Record Projects tool allows end-users within and across organizations to collaborate on project deliverables. For example, end-users might provide feedback on a health promotion video collectively developed or collectively interpret an epidemiological analysis.
